# Human Rotavirus Replicates in Salivary Glands and Primes Immune Responses in Facial and Intestinal Lymphoid Tissues of Gnotobiotic Pigs

**DOI:** 10.3390/v15091864

**Published:** 2023-08-31

**Authors:** Charlotte Nyblade, Peng Zhou, Maggie Frazier, Annie Frazier, Casey Hensley, Ariana Fantasia-Davis, Shabihah Shahrudin, Miranda Hoffer, Chantal Ama Agbemabiese, Lauren LaRue, Mario Barro, John T. Patton, Viviana Parreño, Lijuan Yuan

**Affiliations:** 1 Department of Biomedical Sciences and Pathobiology, Virginia Polytechnic and State University, Blacksburg, VA 24061, USA; charlottejn@vt.edu (C.N.); pengz81@vt.edu (P.Z.); mrf1112@vt.edu (M.F.); atf1012@vt.edu (A.F.); lhcasey@vt.edu (C.H.); aaf222@vt.edu (A.F.-D.); vparreno2021@vt.edu (V.P.); 2 Department of Biology, Indiana University, Bloomington, IN 47405, USA; shashahr@iu.edu (S.S.); mghoffer@iu.edu (M.H.); chagbem@gmail.com (C.A.A.); jtpatton@iu.edu (J.T.P.); 3 GIVAX Inc.—RAVEN at RA Capital Management, Boston, MA 02116, USA; llarue@givax.bio (L.L.); mbarro@givax.bio (M.B.); 4 INCUINTA, IVIT (INTA-Conicet), Hurligham, Buenos Aires 1686, Argentina

**Keywords:** rotavirus, gnotobiotic pigs, salivary glands

## Abstract

Human rotavirus (HRV) is a leading cause of viral gastroenteritis in children across the globe. The virus has long been established as a pathogen of the gastrointestinal tract, targeting small intestine epithelial cells and leading to diarrhea, nausea, and vomiting. Recently, this classical infection pathway was challenged by the findings that murine strains of rotavirus can infect the salivary glands of pups and dams and transmit via saliva from pups to dams during suckling. Here, we aimed to determine if HRV was also capable of infecting salivary glands and spreading in saliva using a gnotobiotic (Gn) pig model of HRV infection and disease. Gn pigs were orally inoculated with various strains of HRV, and virus shedding was monitored for several days post-inoculation. HRV was shed nasally and in feces in all inoculated pigs. Infectious HRV was detected in the saliva of four piglets. Structural and non-structural HRV proteins, as well as the HRV genome, were detected in the intestinal and facial tissues of inoculated pigs. The pigs developed high IgM antibody responses in serum and small intestinal contents at 10 days post-inoculation. Additionally, inoculated pigs had HRV-specific IgM antibody-secreting cells present in the ileum, tonsils, and facial lymphoid tissues. Taken together, these findings indicate that HRV can replicate in salivary tissues and prime immune responses in both intestinal and facial lymphoid tissues of Gn pigs.

## 1. Introduction

Human rotavirus (HRV) is a leading cause of viral-associated gastroenteritis across the globe [1,2]. Following an incubation period ranging from 1 to 3 days, infected individuals develop nausea, diarrhea, and vomiting [1,3]. Infections are typically self-limiting in hosts with competent immune systems; however, infants and immunocompromised individuals are more at risk for severe gastroenteritis and prolonged viral shedding [3,4,5]. The average length of an HRV infection is 5–7 days [2,4]. 

HRV is well established as an enteric pathogen that can spread via the fecal–oral route of infection [3]. Following ingestion of the particles, HRV enters the gastrointestinal tract and infects epithelial cells [3]. HRV targets mature villus enterocytes and enteroendocrine cells while sparing crypt cells [3,6]. Following binding to sialic acids and histoblood group antigens (HBGAs), HRV is endocytosed [6]. The outermost particle layer is discarded, and transcription of viral mRNA begins [6]. Translation, as well as the formation of new double-layered particles (DLPs), occurs in the viroplasm [6]. DLPs bind NSP4 and enter the endoplasmic reticulum, where they finish maturation into triple-layered particles (TLPs) [6]. Viral release occurs via cell lysis, and the replication process continues [6]. Destruction of intestinal cells via lysis is responsible for the malabsorptive component of HRV-induced diarrhea [3,7]. A second arm of HRV-induced diarrhea is secretory. This is moderated in part by the neuro-enterotoxin action of the HRV protein NSP4 and in part by the disruption of intracellular ion levels [3,7]. 

It was recently reported that in addition to replicating in intestinal epithelial cells, murine strains of rotavirus (MRV) could replicate in the salivary glands of pups and dams and transmit via saliva [8]. Orally inoculated pups were able to infect dams via suckling, resulting in an infection of the mammary glands and a spike in secretory IgA production [8]. Furthermore, orally infected adult mice had high titers of virus that were detectable in saliva as early as 3 days post-infection (DPI) [8]. Viral genomes were consistently shed in saliva anywhere from 7 days to 3 weeks post-infection, depending on the strain used for inoculation [8].

The question of whether human strains of enteric viruses can replicate in salivary glands is yet to be determined. The saliva of children infected with HRV shows differential cytokine patterns compared to healthy controls; however, no work has been conducted to evaluate if HRV itself is present in the saliva or salivary glands [9]. To answer this question, we infected gnotobiotic (Gn) pigs with different strains of rotavirus. Gn pigs were selected as the study model due to their human-like gastrointestinal and immune physiology as well as their ability to mimic the symptoms of HRV infection in humans [10,11]. We collected various facial and intestinal tissues to determine the virus’ replicative ability in each one and recorded clinical signs of infection (diarrhea and virus shedding) for all animals.

## 2. Materials and Methods

### 2.1. Study Design

Gn pigs were derived via surgical hysterectomy, housed in germ-free isolators, and fed ultra-high temperature sterilized whole cow’s milk as previously described [12]. Neonatal (5–7 day old) Gn pigs were orally inoculated with 5 × 10^7^ FFU of the attenuated Wa strain of rotavirus (Wa AttHRV), Rotarix (the licensed, live, oral attenuated vaccine from GlaxoSmithKline) [13], or recombinant rhesus rotaviruses (rRRVs) (a component of the formerly available RotaShield vaccine from Wyeth Laboratories) [14] suspended in Diluent #5. From post-inoculation day (PID) 0–7, rectal and nasal swabs (RS and NS) were collected to evaluate diarrhea and virus shedding. At PID2, 1–2 pigs per group were euthanized for evaluation of viral replication in different tissues. Nasal tissue was collected by scraping the interior cavity with a scalpel and transferring it to a microscope slide. Ileum, sublingual, submandibular, and parotid salivary glands were collected in 10% neutral buffered formalin and submitted to the Virginia Tech Animal Laboratory Services (ViTALS) at the Virginia-Maryland College of Veterinary Medicine for embedding in paraffin and sectioning to slides. Sections of all salivary glands plus the salivary duct and ileum were also collected in RNA-Later and frozen at −80 °C until use. At PID10, all remaining rotavirus infected piglets were euthanized to evaluate priming of immune responses. The ileum, tonsils, and facial lymph nodes were collected for detection of rotavirus specific IgM antibody secreting cells (ASCs.) Serum, small intestinal contents (SIC), and large intestinal contents (LIC) were collected for rotavirus specific IgM titers. The division of Gn pigs as well as samples collected for each group is summarized in Supplementary Table S1. 

### 2.2. Virus Preparation

Wa AttHRV inoculum was prepared as described previously [15]. Briefly, the titer of the inoculum stock was determined using a virus ELISPOT [16], and then a total of 5 × 10^7^ FFU of the attenuated virus was diluted in Diluent #5 (minimal essential medium with 1% penicillin-streptomycin and 1% HEPES).

To generate recombinant rotaviruses, the complete simian puc19-RRV plasmid collection was commercially synthesized (Genewiz, South Plainfield, NJ, USA) (HQ846843-HQ846853) under the control of a T7 transcription promoter. The reverse genetics protocol used to generate recombinant rotaviruses was described in detail previously with modifications [17,18]. Briefly, BHK-T7 cell monolayers in 12-well plates were transfected with RRV plasmids and pCMV-NP868R using TransIT-LT1 (Mirus, MIR 2300, Madison, WI, USA) transfection reagent. Transfection mixtures contained 0.8 µg of each of the 11 plasmids—except for puc19/NSP2RRV and puc19/NSP5RRV, which were used at levels 5-fold higher—were incubated in 100 µL of OptiMEM and 44 µL of transfection reagent for 15 min at room temperature. Two days after transfection, the BHK-T7 cells were overseeded with MA104 cells in the presence of trypsin with a final concentration of 0.5 µg/mL [18]. Three days post-overseeding, the BHK-T7/MA104 cell mixture underwent freeze–thaw cycles, and the lysates were clarified by low-speed centrifugation (800× *g*, 5 min). To amplify recombinant viruses present in the lysate, the trypsin concentration in the lysate was adjusted to 10 µg/mL and incubated for 1 h at 37 °C. MA104 cell monolayer in a 6-well plate was infected with 300 µL of the trypsin-treated lysate and incubated at 37 °C in a CO_2_ incubator until all cells had undergone lysis. Recombinant viruses were recovered from clarified cell lysates by plaque isolation on an MA104 cell monolayer. Plaque-isolated viruses were initially grown on MA104 cell monolayers in T-25 flasks. To generate large virus pools for this Gn piglet study, rRRV was amplified in T-175 flasks of Vero cells at a dilution of 1:20. Virus titer was determined with the virus ELISPOT assay, then 5 × 10^7^ FFU of rRRV suspended in 5 mL of Diluent #5 was given orally to each Gn pig.

### 2.3. Rotavirus RT-qPCR

200 µL of RNA was extracted from RS and NS samples using the Quick-RNA Viral Kit (Zymo Research, R1035, Irvine, CA, USA) according to the manufacturer’s instructions. 50 µL of RNA was extracted from 30 mg of tissue (ileum, salivary gland duct, parotid salivary gland, submandibular salivary gland, or sublingual salivary gland) using an RNeasy Kit (Qiagen, 74004, Germantown, MD, USA) according to the manufacturer’s instructions. The target of amplification is an 87 bp fragment of the highly conserved non-structural protein 3 (NSP3) gene. The forward primer is NVP3-FDeg (5′-ACC ATC TWC ACR TRA CCC TC-3′). The reverse primer is NVP3-R1 (5′-GGT CAC ATA ACG CCC CTA TA-3′). The TaqMan Probe is NVP3-Probe (5′-A 6-carboxyfluorescein [FAM] TG AGC ACA ATA GTT AAA AGC TAA CAC TGT CAA-black hole quencher 1 [BHQ1]-3′) [19]. The RT-qPCR assay was performed by using a SensiFast Probe No-ROX one-step Kit (Meridian Bioscience, Bio76005, Cincinnati, OH, USA) according to the manufacturer’s instructions. 4 µL of viral RNA was used as a template in each amplification. The reactions were carried out in the Bio-Rad CFX96 Real-Time C1000TM Thermal Cycler. The linear standard curve was presented with the positive control RNA from 1 × 10^2^ to 1 × 10^7^ copies/µL. Assay efficiency (10(−1/slope)) was calculated from the slope of the standard curve, which is generated by plotting the log copy number versus the cycle threshold (CT) value. Copy numbers were calculated by using the following equation: Copy number (molecules/µL) = [concentration (ng/µL) × 6.022 × 1023 (molecules/mol)]/[length of amplicon × 640 (g/mol) × 109 ng/g) [19].

### 2.4. Detection of Infectious Rotavirus Viral Particles via Virus ELISPOT

50 µL of the sample (rectal swab, nasal swab, or saliva) and 50 µL of serum-free media containing 0.5 µg/mL of trypsin were added to confluent MA104 cells and incubated for 18 h at 37 °C with 5% CO_2_. Plates were fixed with 80% acetone for 15 min and rinsed with PBS for 2 min. Plates were incubated with HRP conjugated nanobody 2KD1 [20] diluted 1:4000 in PBS for 1 h at 37 °C, then washed 3 times with PBST. Plates were developed using TrueBlue peroxidase until spots were dark and read using CTL ImmunoSpot software, https://immunospot.com/immunospot-software.html. The data is presented as fluorescent focus units per mL (FFU/mL).

### 2.5. Immunostaining for Rotavirus in the Nasal Cavity

Cells on nasal cavity scrap slides were fixed with ice-cold 80% acetone for 15 min at room temperature. The acetone was discarded and the slides were air dried at room temperature and then stored at −20 °C until staining. Slides were removed from −20 °C, warmed to room temperature, and rehydrated with PBS. In-house anti-VP6 nanobody 2KD1 [21] conjugated to Alexa 488 was diluted 1:500 in PBS, added to the slide, and incubated for 1 h at 37 °C. Slides were washed 6 times with PBS 0.05% Tween (PBST). Slides were counterstained with DAPI diluted 1:20,000 in PBS for 5 min at room temperature, then washed 6 times with PBST. The slides were mounted with anti-fade media, enveloped with a cover slip, and observed using a BioTek Cytation 5. Representative slides were chosen for further imaging with a Zeiss LSM 800 confocal.

### 2.6. Immunostaining for Rotavirus in Salivary Glands and Ileum Sections

To detect rotavirus in salivary glands, tissue sections were de-paraffinized by washing in Hemo-De (Fisher Scientific, NC0033993, Waltham, MA, USA) 3 times followed by multiple washes in a decreasing ethanol gradient (100%, 100%, 90%, 80%, 70%). Antigen revival was performed by incubating slides overnight at 60 °C in 0.01 mol citrate buffered saline. Slides were washed with distilled water 3 times, then permeabilized with ice-cold methanol for 15 min. Slides were washed twice with PBS 0.1% Triton X (PBSTx) and then blocked using PBS 5% BSA for 1 h at 37 °C. The blocking solution was discarded, and the following procedures were used for the detection of NSP3 or VP6:

To detect NSP3, in-house antibodies were diluted 1:200 in PBS 5% BSA, applied to the slides, and incubated for 1 h at 37 °C. Slides were washed 3 times with PBSTx, then a goat anti-rabbit IgG (H+L) conjugated to Alexa 488 was diluted 1:1000 in PBS 2.5% BSA and incubated for 30 min at 37 °C. Slides were washed 3 times with PBSTx, then counterstained with DAPI diluted 1:20,000 in PBS for 5 min at room temperature. Slides were washed once with PBSTx, then mounted and imaged as described above.

To detect VP6, 2KD1 conjugated to Alexa 488 was diluted 1:500 in PBS 5% BSA, applied to slides, and incubated for 1 h at 37 °C. Slides were washed 3 times with PBSTx, then counterstained with DAPI diluted 1:20,000 in PBS for 5 min at room temperature. Slides were washed once with PBSTx, then mounted and imaged as described above.

### 2.7. Detection of Virus-Specific IgM ASC by ELISPOT

Mononuclear cells were isolated from the ileum, tonsils, and facial lymph nodes [22,23,24]. All cell counts were determined using Trypan Blue (Thermofisher, 15250061, Waltham, MA, USA) and a Cellometer Auto 200 (Nexcelom, Lawrence, MA, USA).

For the ileum, tissues were washed twice with wash media (RMPI with 200 µg/mL of Gentamicin, 10mM HEPES, and 20 µg/mL of Ampicillin) and twice with Ca^2+^ and Mg^2+^ free HBSS. Tissues were cut into fragments and washed twice with Solution A (Ca^2+^ and Mg^2+^ free HBSS with 200 μg/mL gentamicin, 20 μg/mL ampicillin, 40 mM HEPES, 5 mM EDTA, 0.29 mg/mL DTT, and 7% NaHCO_3_). Tissue fragments were cut again and washed twice with Solution C (RPMI-1640 with 8% FBS, 200 μg/mL gentamicin, 20 μg/mL ampicillin, 20 mM HEPES, 5 mM EDTA, 0.29 mg/mL DTT, and 400 U/mL collagenase type II). The digested tissue was ground through 250 µm mesh of sterile steel sieves and the cell suspension was mixed with Isotonic Percoll. Suspensions were centrifuged without brake at 4 °C for 20 min at 1800× *g*. Cell pellets were transferred to new tubes, resuspended with 43% Percoll, underlaid with 70% Percoll, and centrifuged without brake at 4 °C for 30 min at 1800× *g*. Cells at the interface were collected, resuspended in wash media, and pelleted by centrifugation at 4 °C 800× *g* for 15 min. Cells were resuspended with ERPMI (RMPI, 20 mM HEPES, 100 µg/mL Gentamicin, 8% FBS, 2 mM L-glutamine, 0.1 mM nonessential amino acids, 1mM Sodium Pyruvate, 10 µg/mL Ampicillin).

For the tonsils, tissues were placed into sterile steel sieves with 250 µm mesh, rinsed with Ca^2+^ and Mg^2+^ free HBSS, and pushed through the mesh using the plunger of a 30 mL syringe. Tissues were washed several times until clear. The resultant cell suspension was equally distributed among 50 mL centrifuge tubes, underlaid with 10 mL of Ficoll-Paque PREMIUM (Cytiva, 17544202, Marlborough, MA, USA) and centrifuged without brake at room temperature at 1000× *g* for 20 min. The interface was collected, placed into a new 50 mL tube, and covered with 45 mL of HBSS. Cells were centrifuged at 800× *g* for 10 min at room temperature. The supernatant was discarded, and the cells were resuspended in 45 mL of HBSS and centrifuged again at 800× *g* for 10 min at room temperature. The supernatant was discarded, and the cell pellet was resuspended with ERPMI.

For the facial lymph nodes, tissues were placed into sterile steel sieves with 250 µm mesh, rinsed with wash media, and pushed through the mesh using the plunger of a 30 mL syringe. Tissues were washed until clear. The resultant cell suspension was equally distributed among 50 mL centrifuge tubes, topped to 50 mL with wash media, and centrifuged at 1000× *g* for 30 min at 4 °C. The supernatant was discarded, and the pellet was resuspended with 20 mL of wash media. 13.5 mL of isotonic Percoll was added, followed by an additional 11 mL of wash media. The cells were centrifuged at 1200× *g* for 30 min, with no brake at 4 °C. The cell pellet was collected, resuspended in 43% isotonic Percoll, underlaid with 70% isotonic Percoll, and centrifuged at 1900× *g* for 30 min, no brake at 4 °C. The interface was collected and placed into a fresh 50 mL centrifuge tube. 50 mL of wash media was added, and the cell suspension was centrifuged at 504× *g* for 15 min at 4 °C. The supernatant was discarded, and the cell pellet was resuspended in ERPMI. 

For the ELISPOT assay, 5 × 10^5^ MNC were added in duplicate to fixed, Wa AttHRV infected, confluent MA104 cells (CRL-2378.1, ATCC, Manassas, VA, USA) in 96-well plates and incubated for 12 h at 37 °C with 5% CO_2_. Plates were washed 5 times with PBS 0.05% Tween (PBST), then 100 µL per well of biotinylated goat anti-porcine IgM antibody (Bethyl Laboratories, A100-117B, Montgomery, TX, USA) diluted 1:5000 in PBST was added to the plates and incubated for 2 h at room temperature. Plates were washed 5 times with PBST, then incubated with HRP conjugated streptavidin (5270-0029, KPL, Milford, MA, USA) diluted 1:30,000 in PBST for 1 h at room temperature. Plates were washed 5 times with PBST, developed with TMB TrueBlue (5510-0054, KPL, Milford, MA, USA) until spots became dark blue, and read with CTL ImmunoSpot software. Data is presented as ASC#/ 5 × 10^5^ MNC.

### 2.8. Detection of Virus-Specific IgM by Antibody ELISA

Corning 96-well plates (Corning, 9017, Corning, NY, USA) were coated with semi-purified Wa AttHRV for 1 h at 37 °C. Plates were washed 5 times with TBS 0.05% Tween (TBST) and blocked with TBS 5% BSA overnight at 4 °C. Serum, SIC, and LIC samples were four-fold serially diluted in TBST 5% BSA in surrogate 96 deep well plates (Thomas Scientific, 1193A46, Swedesboro, NJ, USA), then 100 µL of prepared sample was added to the reaction plate. Plates were incubated overnight at 4 °C and then washed 5 times with TBST. HRP conjugated goat anti-pig IgM (Bethyl Laboratories, A100-117P, Montgomery, TX, USA) was diluted 1:3000 in TBST 1% BSA and added to the plate for 2 h at room temperature. Plates were washed 5 times with TBST and developed with ABTS peroxidase substrate solution (Seracare, 5120-0032, Milford, MA, USA). The OD was read at 405 nm.

## 3. Results

### 3.1. Nasal and Fecal Human Rotavirus Shedding

To evaluate virus shedding, neonatal Gn pigs were divided into four groups (*n* = 3–6 pigs/group) and orally inoculated with 5 × 10^7^ FFU of either Rotarix, rRRV, Wa AttHRV, or mock inoculated with Diluent #5. Daily rectal and nasal swabs were taken from post-inoculation day (PID) 0–7 to evaluate virus shedding. Metrics to evaluate virus shedding included onset day, duration, and magnitude; a summary of all virus shedding results using RT-qPCR can be found in Table 1.

**Figure 1:**
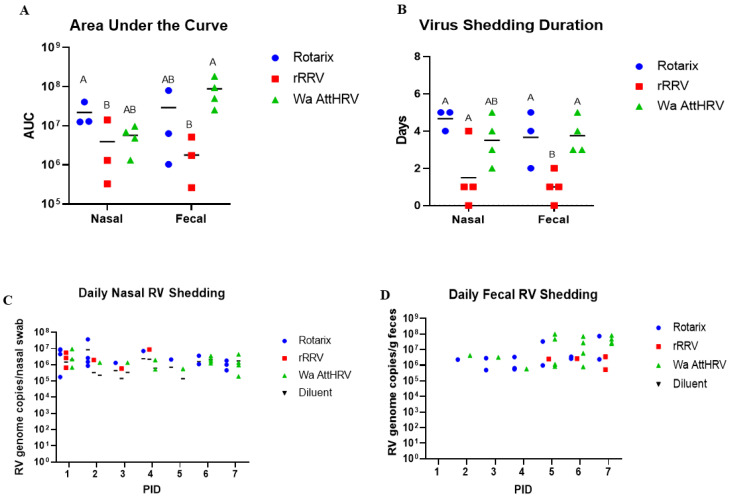
Rotavirus nasal and fecal shedding from PID1-7 in Gn pigs. Gn pigs inoculated with Rotarix, rRRV, or Wa AttHRV shed high levels of rotavirus in feces and nasally (**A**). The duration of virus shedding in both nasal and fecal samples ranged from one to five days (**B**). Genome copies detected in nasal and fecal swabs each day are shown in (**C**,**D**), respectively. Different letters indicate statistical significance, Kruskal–Wallis test followed by Dunn’s multiple comparisons test except Fisher exact test for percentage, *p* < 0.05. AUC: area under the curve.

High levels of virus shedding were detected in nasal and rectal swabs from all inoculated pigs (Figure 1A). In nasal swab samples, the highest viral genome copies, on average, were observed in Rotarix-inoculated pigs, followed by Wa AttHRV and rRRV-inoculated pigs. In fecal samples, the greatest genome copy number was observed in the Wa AttHRV group, then Rotarix and rRRV, respectively. The greatest duration of nasal virus shedding was observed in Rotarix inoculated piglets, with the virus being detected for 4.7 days on average (Figure 1B). Wa AttHRV-inoculated pigs had the second longest duration of nasal virus shedding, with the virus being detected for 3.5 days on average (Figure 1B). The duration of fecal virus shedding was highest for Wa attHRV-inoculated piglets but was closely followed by Rotarix-inoculated animals (3.75 and 3.67 days, respectively) (Figure 1B). rRRV inoculated pigs had the shortest duration of nasal and fecal virus shedding, with nasal shedding observed for 1.5 days on average and fecal shedding for 1 day on average (Figure 1B). Nasal shedding began earlier than fecal shedding, with the virus being detected at PID1 across all groups (Figure 1C). Fecal shedding was detected beginning at PID2 for pigs in the Rotarix and Wa AttHRV groups and at PID5 for pigs inoculated with rRRV (Figure 1D). We also evaluated if the virus in fecal and nasal swabs was infectious using a virus ELISPOT assay [16]. No infectious particles were detected in fecal samples; however, two pigs in the Rotarix and rRRV groups and three pigs in the Wa AttHRV groups had infectious viruses in nasal secretions. A pig-by-pig breakdown of the virus-shedding results is presented in Supplementary Table S2. 

### 3.2. Presence of Human Rotavirus in Salivary Glands and Intestines

**Figure 2:**
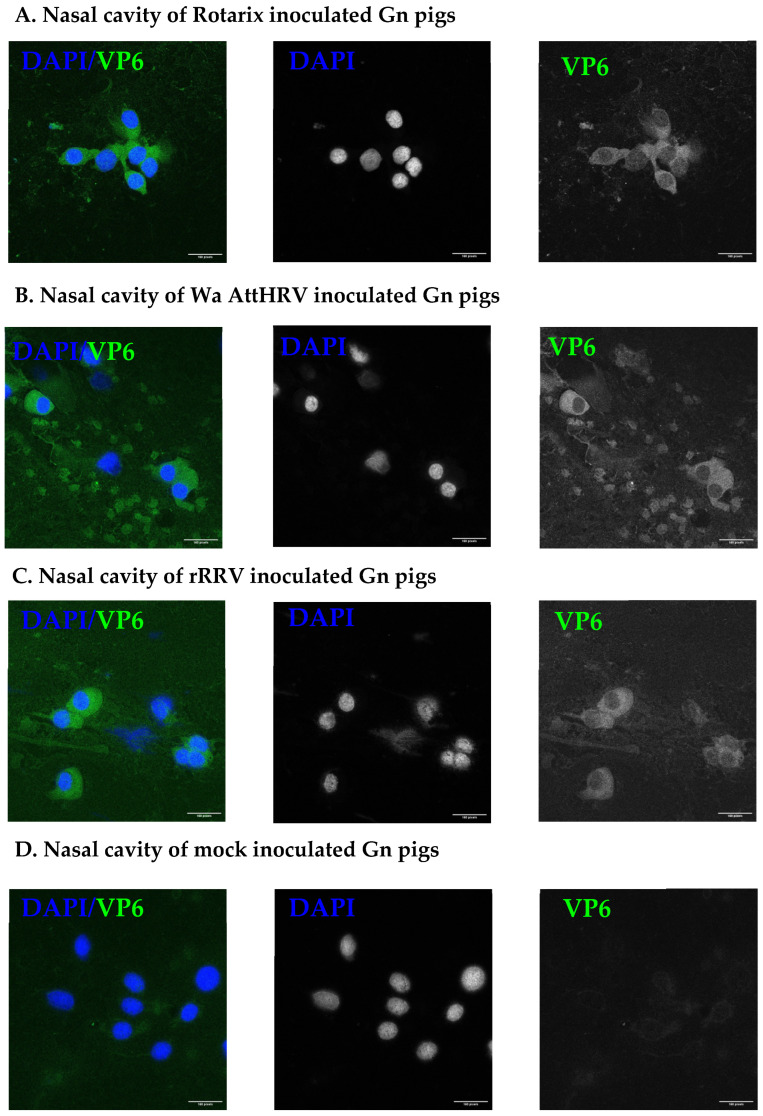
Detection of rotavirus antigen in the nasal cavity of Rotarix (**A**), Wa AttHRV (**B**), rRRV (**C**), or mock (**D**) inoculated pigs. Representative images from each group are shown. Images were taken using 63× objective on Zeiss LSM 880 confocal. Blue: DAPI. Green: rotavirus VP6. Scale bar is 160 pixels.

**Figure 3:**
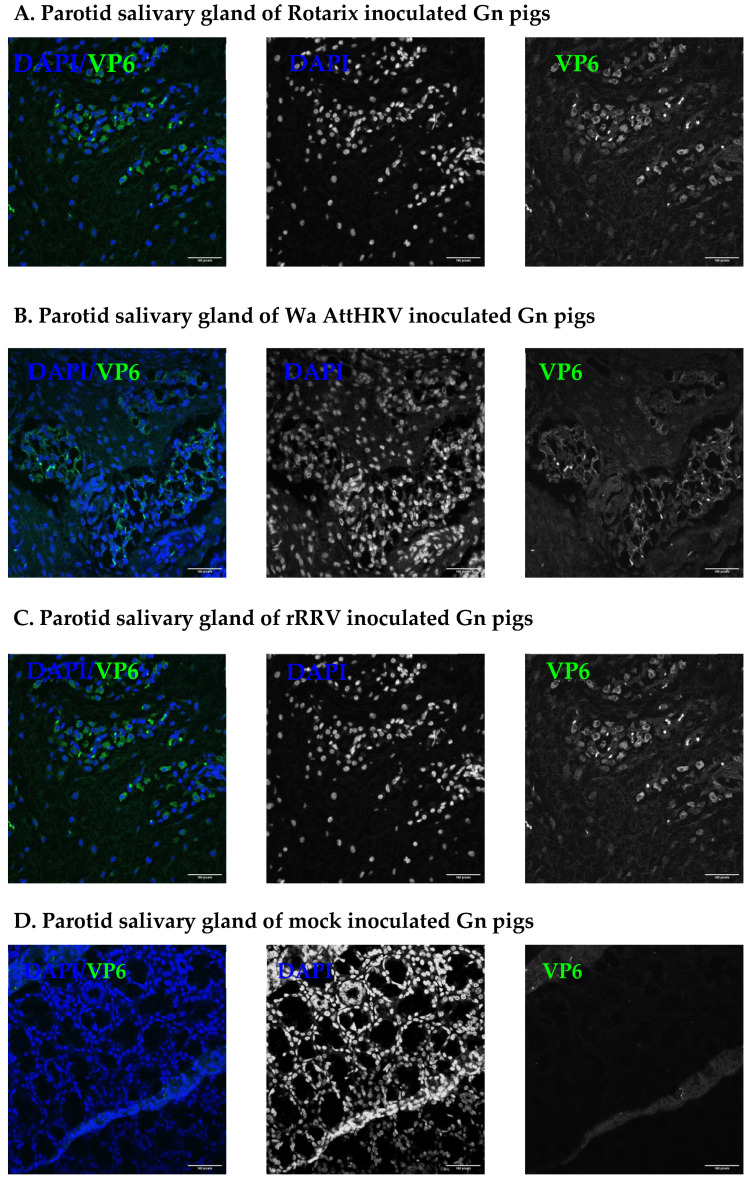
Detection of rotavirus VP6 antigen in the parotid salivary gland of Rotarix (**A**), Wa AttHRV (**B**), rRRV (**C**), or mock (**D**) inoculated pigs. Representative images from each group are shown. Images were taken using 25× objective on Zeiss LSM 880 confocal. Blue: DAPI. Green: rotavirus VP6. Scale bar is 160 pixels.

**Figure 4:**
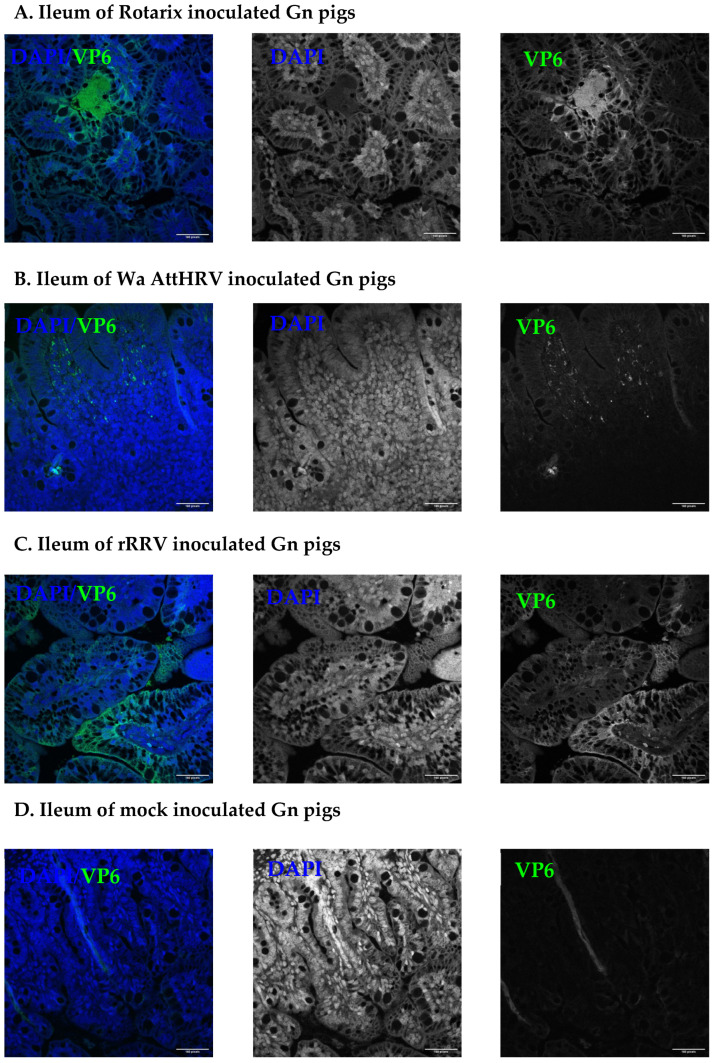
Detection of rotavirus VP6 antigen in the ileum of Rotarix (**A**), Wa AttHRV (**B**), rRRV (**C**), or mock (**D**) inoculated pigs. Representative images from each group are shown. Images were taken using 25× objective on Zeiss LSM 880 confocal. Blue: DAPI. Green: rotavirus VP6. Scale bar is 160 pixels.

At PID2, a subset of pigs inoculated with HRV strains were euthanized for the detection of virus in intestinal and facial tissues. The ileum, parotid salivary gland, submandibular salivary gland, sublingual salivary gland, and nasal cavity were all collected for evaluation (Supplementary Table S2). Rotavirus antigen was detected in multiple tissues of inoculated pigs. In the nasal cavity, rotavirus VP6 was detected in all inoculated pigs (Figure 2). Of the salivary glands collected, rotavirus VP6 was detected in the parotid gland of all inoculated pigs (Figure 3). Only two pigs, one in the Rotarix and one from the Wa AttHRV group, had detectable virus in the submandibular glands. The sublingual glands were negative for the virus. The salivary gland duct of one pig in the Rotarix group was RT-qPCR positive (Supplementary Figure S1). In the ileum of inoculated pigs, viral antigen was widely dispersed across the tissue (Figure 4). All but one pig in the Rotarix group had virus detected in 4 sections of evaluated tissue. High HRV genome copy numbers (ranging from 6.36 × 10^6^ to 7.72 × 10^8^/g tissues) were detected from the ileum of all but one rRRV inoculated pigs (Supplementary Figure S1). Salivary glands and ileums that were positive for VP6 were also positive for NSP3 (Supplementary Figures S2–S4). Lower magnification images for salivary glands are presented in Supplementary Figure S3 to show where the sections within the salivary glands were imaged from. NSP3 staining was observed at similar locations and intensity within the tissue, which was indicative of active viral replication. Due to a limited amount of collected slides, NSP3 staining was not performed for the nasal cavity. A complete pig-by-pig summary of viral antigen detection in tissues is shown in Supplementary Table S2.

### 3.3. Infectious Human Rotavirus Particles in Saliva

Since virus was present in the salivary glands of Gn pigs, we wanted to determine if the virus could also be detected in saliva. Gn pigs were orally inoculated with 5 × 10^7^ FFU of various recombinant rotaviruses (*n* = 2/group) or Diluent #5 (*n* = 1) and euthanized at PID2, where saliva was collected by swabbing the mouth of piglets with cotton swabs and mixing the swabs with 1 mL of sterile PBS. Low numbers of infectious viral particles were detected in the saliva of four pigs by virus ELISPOT. Three out of four pigs had titers of 10 FFU/mL in saliva. The remaining one piglet had a salivary virus titer of 40 FFU/mL.

### 3.4. Detection of Rotavirus-Specific IgM Antibodies and IgM Antibody Secreting Cells

**Figure 5:**
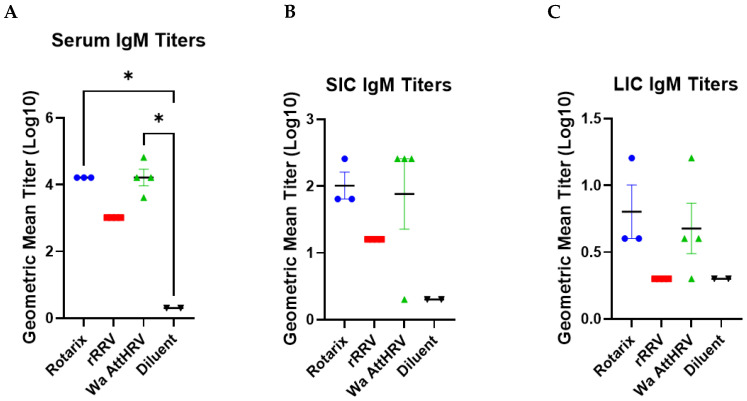
Rotavirus-specific IgM antibody titers in serum (**A**), small intestinal contents (SIC) (**B**), and large intestinal contents (LIC) (**C**). Error bars indicate standard error of the mean. Kruskall–Wallis test for multiple comparisons. * *p* < 0.05.

At euthanasia, the serum, SIC, and LIC were collected and assessed for virus-specific IgM titers. Rotarix, rRRV, and Wa AttHRV inoculated pigs had high titers of systemic and intestinal rotavirus-specific IgM antibodies (Figure 5). In the serum, Rotarix- and Wa AttHRV-inoculated animals had comparable titers of IgM (Figure 5A). This trend was also observed for IgM titers in the SIC and LIC (Figure 5B,C). IgM responses induced by rRRV were lower than Rotarix and Wa AttHRV in serum and SIC and were absent in LIC.

**Figure 6:**
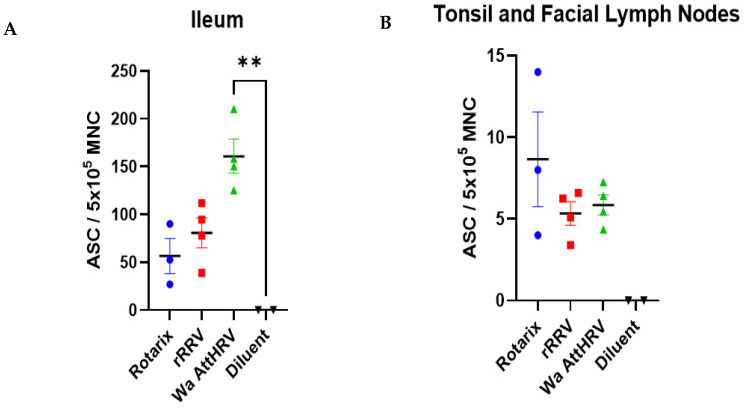
Rotavirus-specific IgM antibody-secreting cells detected in the ileum (**A**), tonsils (**B**), and facial lymph nodes (**B**) of Gn pigs. Error bars indicate standard error of the mean. Kruskall–Wallis test for multiple comparisons. ** *p* < 0.01.

Mononuclear cells were isolated from the tonsils, facial lymph nodes, and ileum of all pigs. Due to low cell counts, the isolated cells from the tonsils and the facial lymph nodes were combined. There was an overall greater magnitude of IgM ASC in the ileum of infected pigs as compared to those in the tonsils and facial lymph nodes (note the different y-axis scales in Figure 6). In the ileum, Wa AttHRV-infected pigs had the highest number of ASCs, followed by rRRV and Rotarix-inoculated pigs (Figure 6A). In the tonsils and facial lymph nodes, Rotarix-inoculated animals had the greatest number of ASCs (Figure 6B). rRRV- and Wa AttHRV-inoculated animals had similar mean numbers of ASCs. 

## 4. Discussion

In this study, we evaluated the ability of HRV to replicate in facial and intestinal tissues. Both fecal and nasal swab samples were positive for rotavirus. 50–80% of inoculated piglets shedding rotavirus by PID2. Only two piglets in the Wa AttHRV group, none in the rRRV group, developed diarrhea following inoculation, indicating the rRRV is a fully attenuated rotavirus. Piglets orally inoculated with rotaviruses shed virus nasally for up to 5 days, with an average onset day ranging from 1–3 across groups. The onset of nasal shedding was similar to that seen in a study by Azevedo et al., who observed the onset day of attenuated or virulent Wa HRV nasal shedding to be between 1.6 and 2.4 [25]. The duration of nasal shedding observed in our pigs was fairly similar as well. Nasal rRRV shedding lasted 1.5 days on average, nearly identical to the 1.4 days of attenuated HRV shedding seen by Azevedo [25]. However, Wa AttHRV-inoculated pigs had nasal shedding durations that more closely matched the nasal shedding pattern of pigs orally inoculated with virulent Wa HRV (3.5 versus 3.4 days) rather than the attenuated strain (1.6 days). The duration of nasal Rotarix shedding was closest to piglets intranasally inoculated with virulent Wa HRV (4.67 versus 5 days) [25]. Nasal viral titers in rRRV, Wa AttHRV, and Rotarix inoculated pigs were significantly higher than those observed by Azevedo et. al. These differences could be attributed to sensitivity differences in detector assays used between groups (virus ELISPOT and RT-qPCR compared to ELISA and cell culture immunofluorescence). 

At 2 days post-infection, we found viral antigen in the ileum, nasal cavity, and salivary glands of Rotarix-, Wa AttHRV-, and rRRV-inoculated Gn pigs. Detection of virus at this time point is consistent with the observations of murine enteric virus replication in salivary glands [8]. We also detected infectious viral particles in the saliva of four pigs. The length of viral replication and shedding in the salivary gland complex remains to be elucidated. When evaluating the duration of murine norovirus and rotavirus infection in salivary glands, acute infection strains MNV-1 and EDIM were cleared from saliva within 7–10 days, whereas persistent strains MNV-3, MNV-4, and WU23 could be detected up to three weeks post-inoculation [8]. As our animals were euthanized at early time points only, it was not possible to determine if HRV is persistently shed in saliva or only a temporary part of acute infection. Future studies can follow animals for a greater time to quantify if the length of infectious virus spread from salivary glands follows a similar pattern to that of virus shed in feces and nasal secretions. Elucidating these details will be important for developing public health interventions to curb virus spread, such as methods to inhibit transmission via aerosolization and durations of quarantines.

Evaluating if strain differences influence salivary gland tissue localization is also one future direction for this work. In this study, we used G1P[8] (Rotarix and Wa AttHRV) and G3P[3] (rRRV) strains of HRV. Variations between the number of infected issues could be due to sampling methods, such as a low animal number and limited tissue sections, or due to intrinsic differences between strains (G- and P-type). In a murine model of infection, the CR6 strain of norovirus was unable to infect salivary glands, standing in contrast to the MNV1, 3, 4, and WU23 strains [8]. This hints at strain-unique differences in infection. While evaluating strain-specific differences is beyond the scope of this paper, a future study could elucidate this question by using higher numbers of animals as well as additional strains of HRV that are prevalent across the globe. The globally dominant HRV genotypes include P[8], P[4], and P[6], with others being detected occasionally; rotavirus infectivity in vivo is in a P genotype-dependent manner [26]. Determining if several P types or only a select few Group A rotaviruses can replicate in salivary glands would be useful information for shaping the vaccine development pipeline, as vaccine formulation could be modified to specifically target buccal or nasal cavity surfaces as opposed to a parenteral administration.

We only evaluated IgM responses in order to assess the ability of the viruses to prime antibody responses within the time frame of the short experiment. Future studies should also evaluate IgA and IgG responses. Infection with all three rotaviruses induced strong systemic and intestinal virus-specific IgM antibody responses, with antibodies being detected in serum, SIC, and LIC. The lower IgM titers and ASC numbers in rRRV-inoculated pigs relative to Wa AttHRV- and Rotarix-inoculated animals are likely due to the heterotypic nature of the coating antigen (Wa AttHRV) used in the assays. The ability of the rRRV inoculum to prime immune responses, much like Rotarix and Wa AttHRV, is an exciting find, as it has been demonstrated that mucosal immunity to HRV is crucial for protection against infection and disease [3]. The rRRV and Rotarix have the capacity to serve as a backbone for a dual HRV-HuNoV vaccine, as the usage of reverse genetics systems enables recombinant rotaviruses to express the proteins of other viruses [27]. Data from a study with immunocompetent and *Stat1*^−/−^ suckling mouse models showed rRRV and rRRV-expressing HuNoV VP1 induced diarrhea and virus shedding, with immunocompetent mice having more modest virus shedding patterns than *Stat1* knockouts [28]. Furthermore, following an initial oral inoculation and booster intraperitoneal injection at 9 weeks post-inoculation, both mouse groups developed HuNoV VP1-specific serum IgG and fecal IgA responses [28]. While promising as proof of principle experiments, additional preclinical work assessing the immunogenicity of rRRV and rRRV expressing HuNoV proteins in more human-like animal models is necessary before moving further with vaccine development. This includes quantifying humoral and cell-mediated immune responses at systemic, intestinal, and facial lymphoid tissue sites following oral booster doses and performing viral challenge studies with HRV, HuNoV, or both pathogens to determine protective efficacy.

The induction of IgM antibody responses in Wa AttHRV, rRRV, and Rotarix inoculated pigs was further supported by the detection of ASCs in intestinal and facial lymphoid tissues of the Gn pigs. ASC cell numbers in the ileum of all rotavirus-inoculated pigs were high, ranging from 56 to 161 ASC/ 5 × 10^5^ MNC. Rotavirus inoculations induced ASC responses in facial lymphoid tissues as well, though to a lesser degree relative to the ileum. These two findings indicate immunity is being primed at the sites of infection. A limitation of this study is the tonsil and facial lymph node MNC were combined due to low numbers of cells, and thus we are unable to tell if these immune responses are skewed to one tissue or equally distributed among both. Moving forward, we plan to separate these cells based on tissue to evaluate where the ASCs are concentrated. The usage of older animals with larger tissue sections for digestion will aid in this process.

Elucidating other components of saliva following HRV infection will also be important moving forward. For example, determining the cytokine profiles in saliva collected from infected versus non-infected pigs. Human children at an acute stage of HRV infection exhibit differential cytokine levels compared to both children at a convalescent stage (90 days post-infection) and healthy controls [9]. Pro-inflammatory cytokines IL-8, IL1-β, and TNFα were all increased compared to controls [9]. The most significantly upregulated cytokine was IL-22, a cytokine primarily secreted by immune cells [9]. Infected Gn pigs may mimic these cytokine increases. Their saliva may also contain other correlates of protection. For example, epidermal growth factor (EGF) or activated T cells. EGF is a protein important for the maintenance of epithelial cell integrity, and HRV-infected children have increased levels of it in their saliva at an acute stage of infection [29]. This increase was associated with less severe infections as determined by shorter hospital stays [29]. Multiple studies have shown that T cells are activated by HRV infection and these cells can support the humoral immune response to promote infection clearance [30]. The detection of CD4+ or CD8+ T cells in saliva would likely coincide with changes to cytokine profiles and help build the story of rotavirus infection in salivary glands.

## 5. Conclusions

In summary, we found evidence that HRV replicates in salivary gland and intestinal tissues and induces immune responses in intestinal and facial lymphoid tissues. This challenges the classical pathway of fecal–oral transmission of this virus and may influence the design of vaccines, methods for controlling viral spread, and a general understanding of tissue tropism of enteric viruses.

## Figures and Tables

**Table 1:** Summary of rotavirus shedding in Gn pigs by RT-qPCR. Different capital letters indicate statistical significance. No letters or shared letters indicate no difference. Values in parenthesis are standard errors of the mean. Kruskal–Wallis test followed by Dunn’s multiple comparisons test except Fisher exact test for percentage, *p* < 0.05. AUC: area under the curve.

	Nasal				
Group	%Shedding Virus	Mean Days to Onset	Mean Duration	Mean Peak Titer	AUC Virus Shedding
**Rotarix**	4/5 (80%) ^A^	1.0 (0) ^A^	4.67 (0.33) ^A^	1.81 × 10^7^ (9.5 × 10^6^)	1.32 × 10^7^ (7.41 × 10^6^) ^A^
**rRRV**	3/6 (50%) ^AB^	1.5 (0.5) ^A^	1.5 (0.87) ^B^	3.0 × 10^6^ (2.0 × 10^6^)	2.4 × 106 (2.34 × 106) ^B^
**Wa AttHRV**	4/6 (66.7%) ^AB^	3.0 (1.23) ^A^	3.5 (0.65) ^AB^	4.61 × 10^6^ (1.69 × 10^6^)	3.78 × 10^6^ (1.63 × 10^6^) ^AB^
**Diluent**	0/1 ^B^	NA	0	0	0
	**Fecal**				
**Rotarix**	4/5 (80%) ^A^	1.0 (0) ^B^	3.67 (0.88) ^A^	2.63 × 10^7^ (2.41 × 10^7^)	1.77 × 10^7^ (1.56 × 10^7^) ^AB^
**rRRV**	3/6 (50%) ^AB^	1.5 (0.5) ^A^	1.0 (0.41) ^B^	1.67 × 10^6^ (8.37 × 10^5^)	1.19 × 10^6^ (8.39 × 10^5^) ^B^
**Wa AttHRV**	4/6 (66.7%) ^AB^	3.0 (1.23) ^B^	3.75 (0.48) ^A^	6.44 × 10^7^ (1.64 × 10^7^)	5.89 × 10^7^ (2.88 × 10^7^) ^A^
**Diluent**	0/1 ^B^	NA	0	0	0

## Data Availability

All relevant data are included in the manuscript.

## Supplementary Materials

The following supporting information can be downloaded at https://www.mdpi.com/article/10.3390/v15091864/s1. Table S1: Division of Gn pigs; Table S2: Detection of viral genomes (by RT-qPCR), infectious viral particles (by virus ELISPOT), and viral antigen (by immunofluorescent staining) in individual pigs infected with rotavirus strains of diluent; Figure S1: Rotavirus genome detection in ileum and salivary gland tissue; Figure S2: Detection of rotavirus NSP3 antigen in the parotid salivary gland of Rotarix (A), Wa AttHRV (B), rRRV (C), or mock (D) inoculated pigs. Images were taken using 25× objective on Zeiss LSM 880 confocal. Blue: DAPI. Green: rotavirus VP6; Figure S3: Detection of rotavirus VP6 or NSP3 antigen in the parotid salivary gland of Rotarix (A), Wa AttHRV (B), rRRV (C), or mock (D) inoculated pigs. Images were taken at 10× on a BioRad Cytation 5. Blue: DAPI. Green: VP6 or NSP3; Figure S4: Detection of rotavirus NSP3 antigen in the ileum of Rotarix (A), Wa AttHRV (B), rRRV (C), or mock (D) inoculated pigs. Images were taken using 25× objective on Zeiss LSM 880 confocal. Blue: DAPI. Green: NSP3.
