# Supplementary material for: Human Rotavirus Replicates in Salivary Glands and Primes Immune Responses in Facial and Intestinal Lymphoid Tissues of Gnotobiotic Pigs
Source: Viruses. 2023 Aug 31;15(9):1864. doi: 10.3390/v15091864 (PMC10534682; doi:10.3390/v15091864)
Supplement: Supplementary file 1 [file viruses-15-01864-s001.zip › viruses-2587275-supplementary.pptx]

## Slide 1
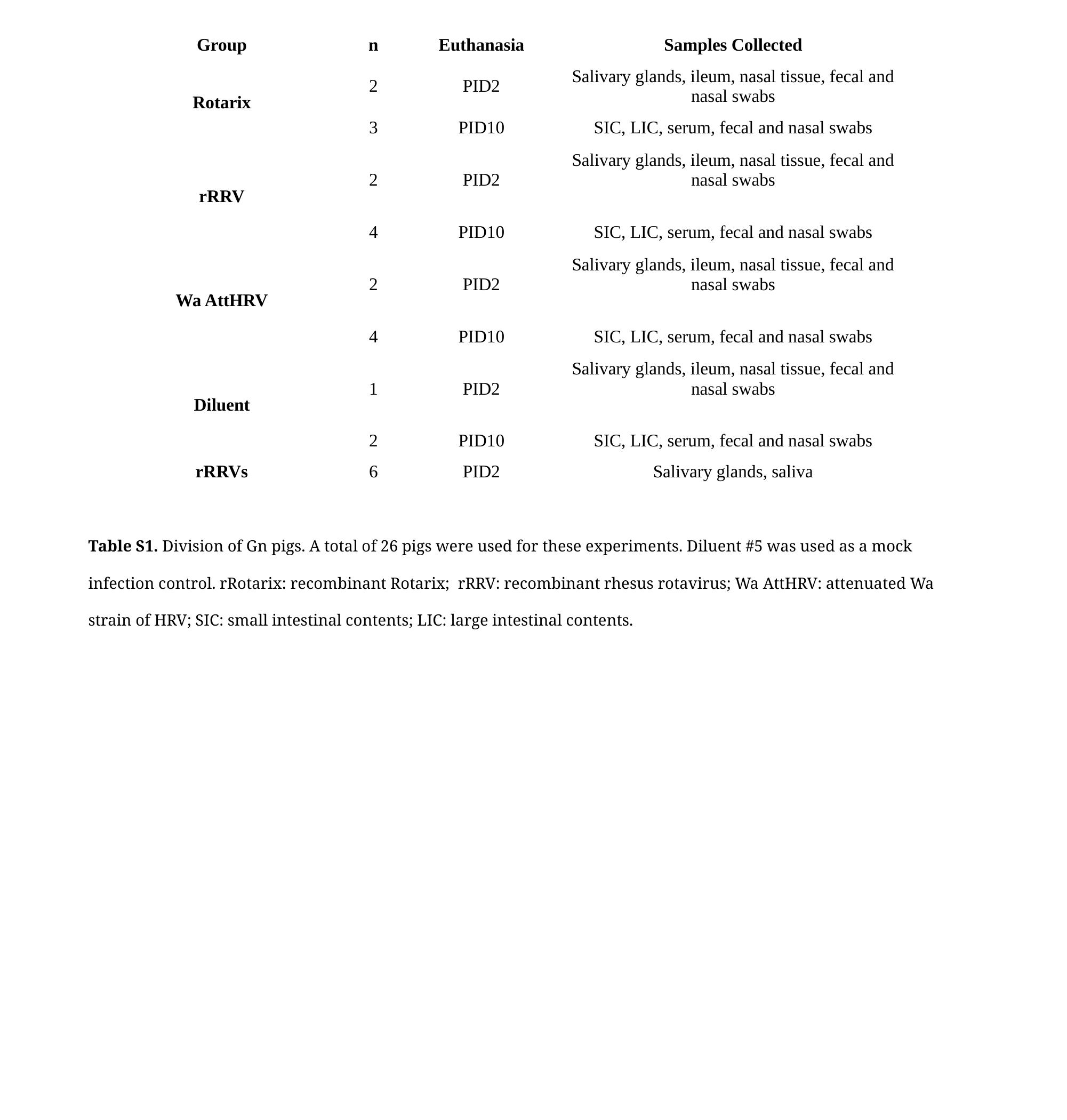

| Group | n | Euthanasia | Samples Collected |
| --- | --- | --- | --- |
| Rotarix | 2 | PID2 | Salivary glands, ileum, nasal tissue, fecal and nasal swabs |
| | 3 | PID10 | SIC, LIC, serum, fecal and nasal swabs |
| rRRV | 2 | PID2 | Salivary glands, ileum, nasal tissue, fecal and nasal swabs |
| | 4 | PID10 | SIC, LIC, serum, fecal and nasal swabs |
| Wa AttHRV | 2 | PID2 | Salivary glands, ileum, nasal tissue, fecal and nasal swabs |
| | 4 | PID10 | SIC, LIC, serum, fecal and nasal swabs |
| Diluent | 1 | PID2 | Salivary glands, ileum, nasal tissue, fecal and nasal swabs |
| | 2 | PID10 | SIC, LIC, serum, fecal and nasal swabs |
| rRRVs | 6 | PID2 | Salivary glands, saliva |
Table S1. Division of Gn pigs. A total of 26 pigs were used for these experiments. Diluent #5 was used as a mock infection control. rRotarix: recombinant Rotarix; rRRV: recombinant rhesus rotavirus; Wa AttHRV: attenuated Wa strain of HRV; SIC: small intestinal contents; LIC: large intestinal contents.

## Slide 2
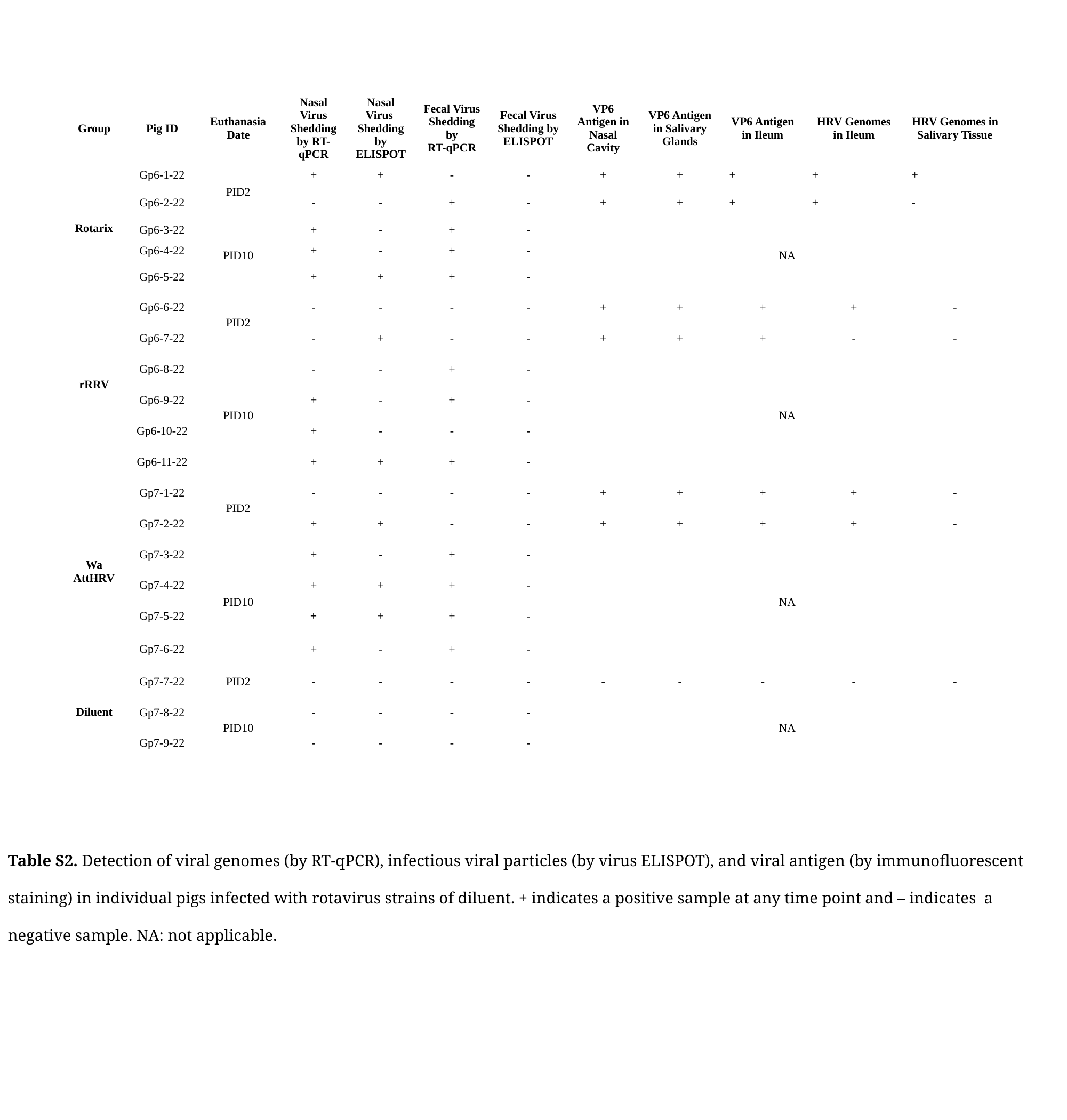

| Group | Pig ID | Euthanasia Date | Nasal Virus Shedding by RT-qPCR | Nasal Virus Shedding by ELISPOT | Fecal Virus Shedding by RT-qPCR | Fecal Virus Shedding by ELISPOT | VP6 Antigen in Nasal Cavity | VP6 Antigen in Salivary Glands | VP6 Antigen in Ileum | HRV Genomes in Ileum | HRV Genomes in Salivary Tissue |
| --- | --- | --- | --- | --- | --- | --- | --- | --- | --- | --- | --- |
| Rotarix | Gp6-1-22 | PID2 | + | + | - | - | + | + | + | + | + |
| | Gp6-2-22 | | - | - | + | - | + | + | + | + | - |
| | Gp6-3-22 | PID10 | + | - | + | - | NA | | | | |
| | Gp6-4-22 | | + | - | + | - | | | | | |
| | Gp6-5-22 | | + | + | + | - | | | | | |
| rRRV | Gp6-6-22 | PID2 | - | - | - | - | + | + | + | + | - |
| | Gp6-7-22 | | - | + | - | - | + | + | + | - | - |
| | Gp6-8-22 | PID10 | - | - | + | - | NA | | | | |
| | Gp6-9-22 | | + | - | + | - | | | | | |
| | Gp6-10-22 | | + | - | - | - | | | | | |
| | Gp6-11-22 | | + | + | + | - | | | | | |
| Wa AttHRV | Gp7-1-22 | PID2 | - | - | - | - | + | + | + | + | - |
| | Gp7-2-22 | | + | + | - | - | + | + | + | + | - |
| | Gp7-3-22 | PID10 | + | - | + | - | NA | | | | |
| | Gp7-4-22 | | + | + | + | - | | | | | |
| | Gp7-5-22 | | + | + | + | - | | | | | |
| | Gp7-6-22 | | + | - | + | - | | | | | |
| Diluent | Gp7-7-22 | PID2 | - | - | - | - | - | - | - | - | - |
| | Gp7-8-22 | PID10 | - | - | - | - | NA | | | | |
| | Gp7-9-22 | | - | - | - | - | | | | | |
Table S2. Detection of viral genomes (by RT-qPCR), infectious viral particles (by virus ELISPOT), and viral antigen (by immunofluorescent staining) in individual pigs infected with rotavirus strains of diluent. + indicates a positive sample at any time point and – indicates a negative sample. NA: not applicable.

## Slide 3
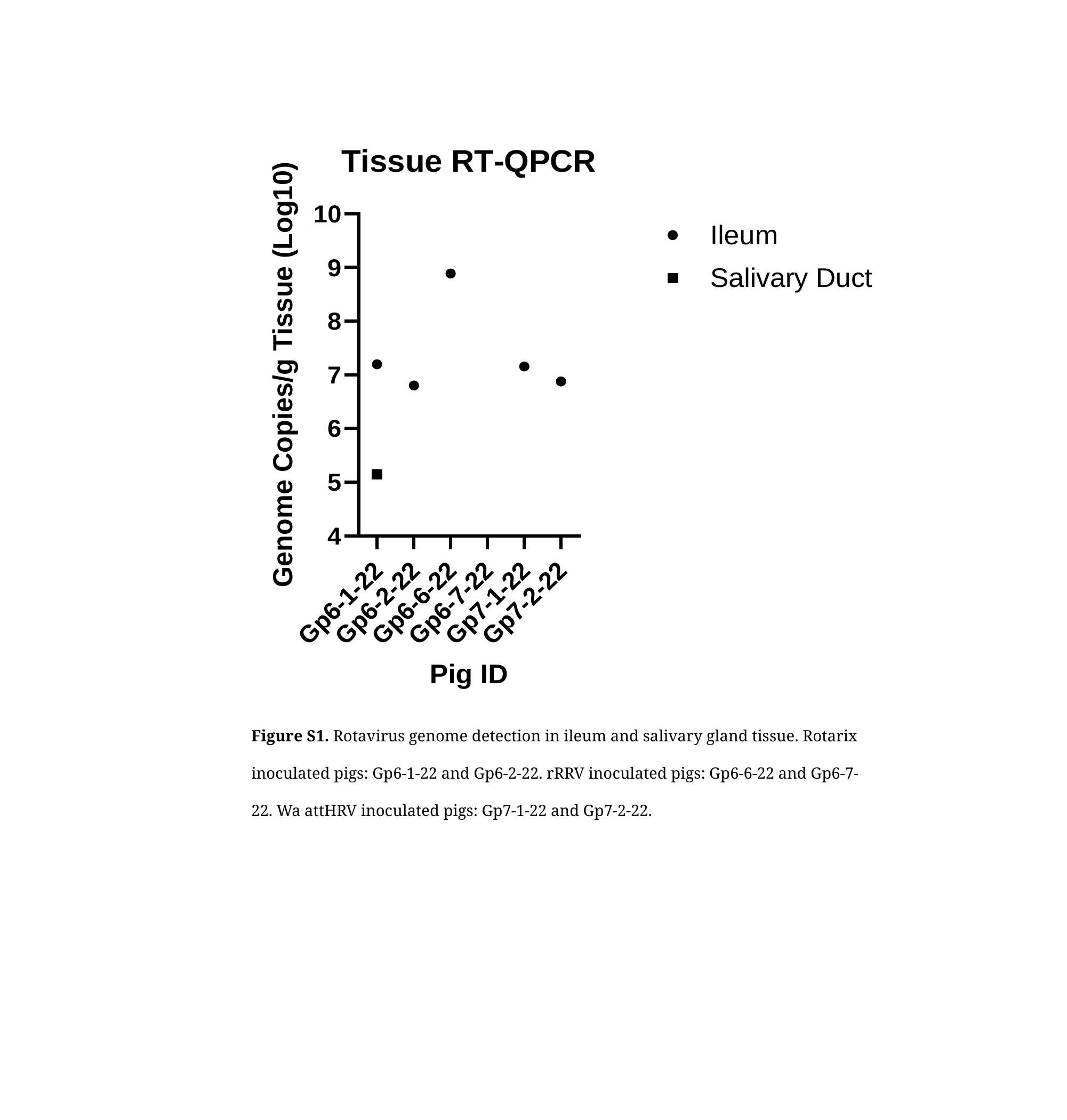

Figure S1. Rotavirus genome detection in ileum and salivary gland tissue. Rotarix inoculated pigs: Gp6-1-22 and Gp6-2-22. rRRV inoculated pigs: Gp6-6-22 and Gp6-7-22. Wa attHRV inoculated pigs: Gp7-1-22 and Gp7-2-22.

## Slide 4
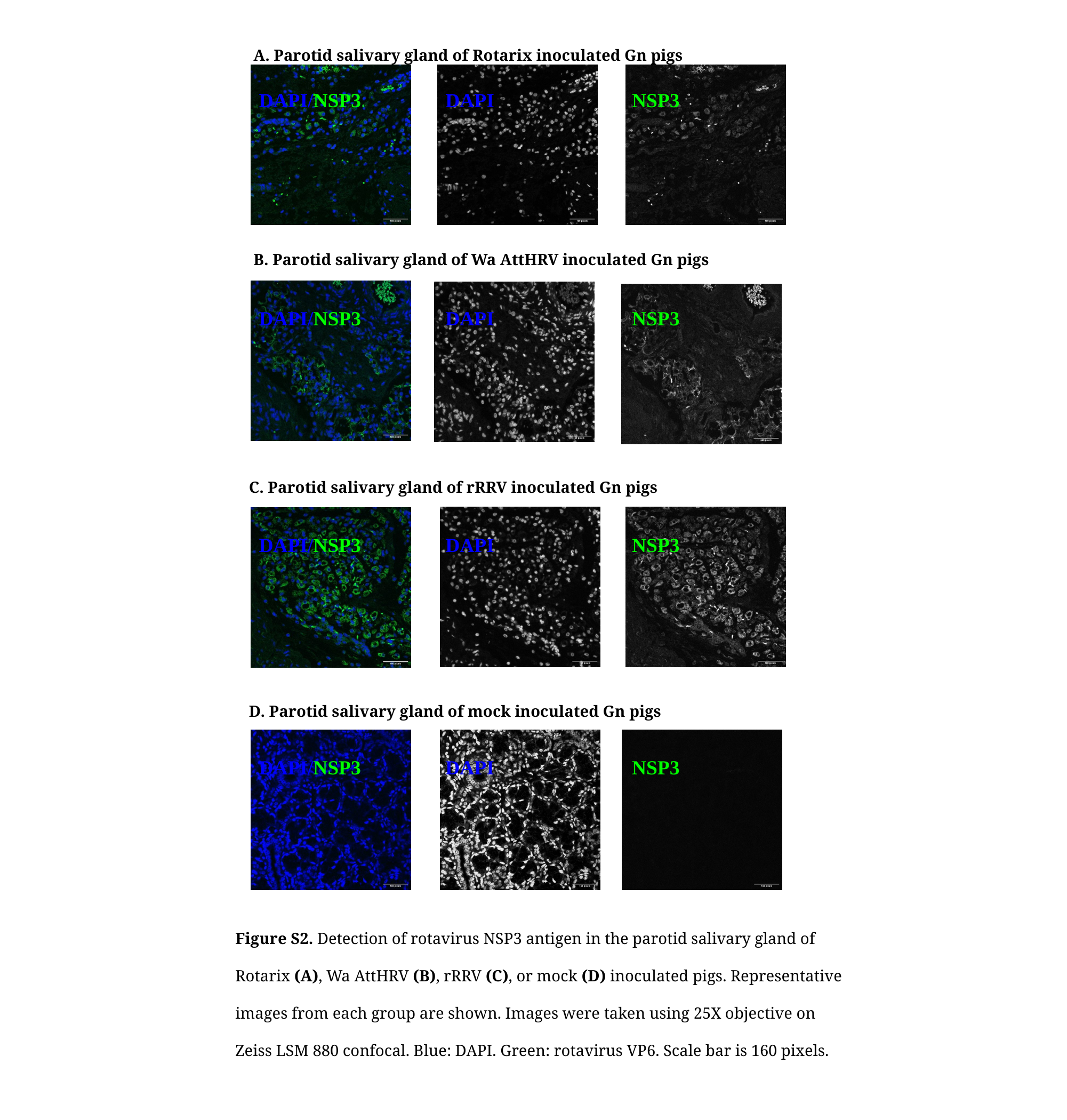

A. Parotid salivary gland of Rotarix inoculated Gn pigs
DAPI/NSP3
DAPI
NSP3
B. Parotid salivary gland of Wa AttHRV inoculated Gn pigs
DAPI/NSP3
DAPI
NSP3
C. Parotid salivary gland of rRRV inoculated Gn pigs
DAPI/NSP3
DAPI
NSP3
D. Parotid salivary gland of mock inoculated Gn pigs
DAPI/NSP3
DAPI
NSP3
Figure S2. Detection of rotavirus NSP3 antigen in the parotid salivary gland of Rotarix (A), Wa AttHRV (B), rRRV (C), or mock (D) inoculated pigs. Representative images from each group are shown. Images were taken using 25X objective on Zeiss LSM 880 confocal. Blue: DAPI. Green: rotavirus VP6. Scale bar is 160 pixels.

## Slide 5
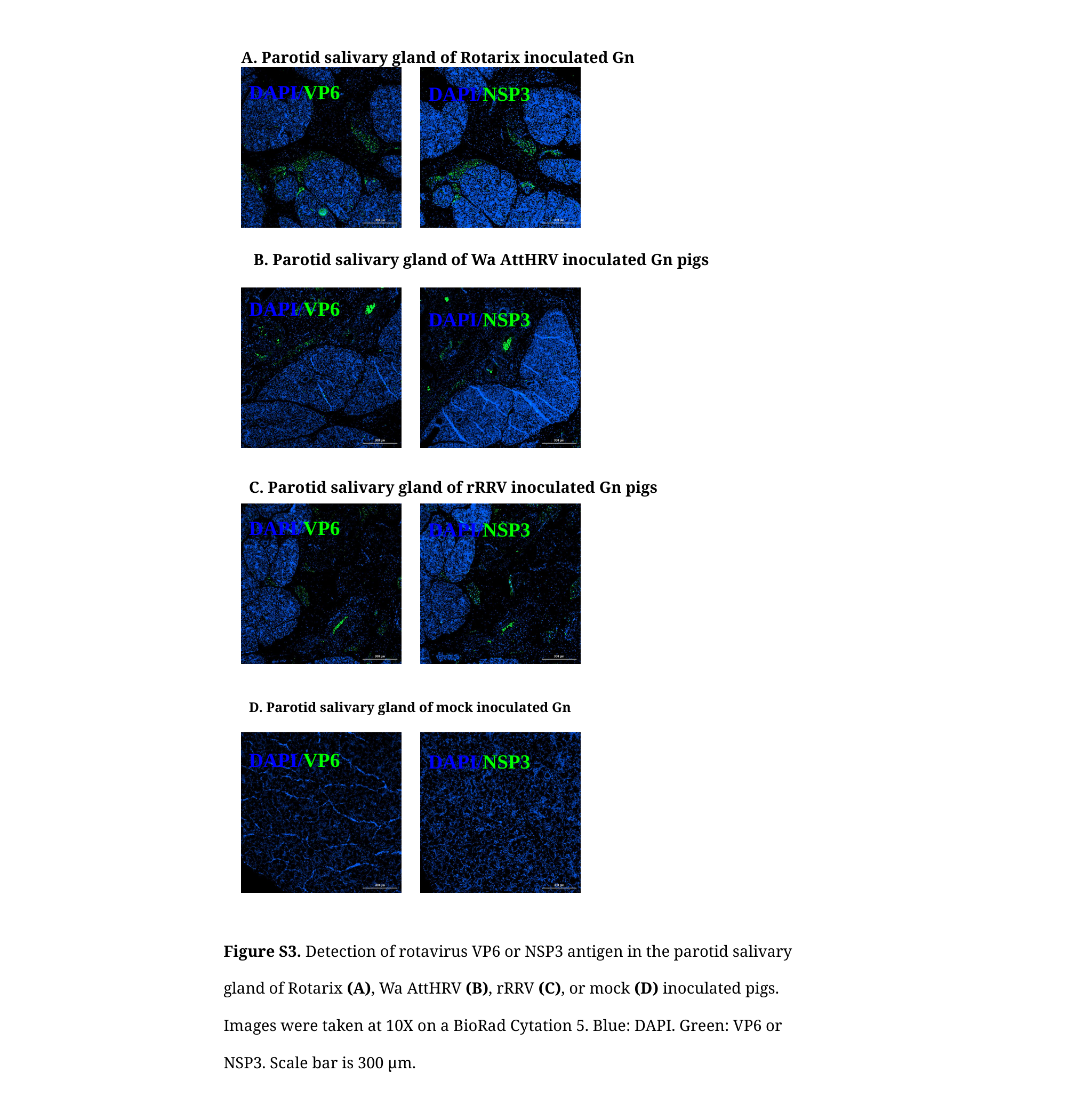

A. Parotid salivary gland of Rotarix inoculated Gn pigs
DAPI/VP6
DAPI/NSP3
B. Parotid salivary gland of Wa AttHRV inoculated Gn pigs
DAPI/VP6
DAPI/NSP3
C. Parotid salivary gland of rRRV inoculated Gn pigs
DAPI/VP6
DAPI/NSP3
D. Parotid salivary gland of mock inoculated Gn pigs
DAPI/VP6
DAPI/NSP3
Figure S3. Detection of rotavirus VP6 or NSP3 antigen in the parotid salivary gland of Rotarix (A), Wa AttHRV (B), rRRV (C), or mock (D) inoculated pigs. Images were taken at 10X on a BioRad Cytation 5. Blue: DAPI. Green: VP6 or NSP3. Scale bar is 300 µm.

## Slide 6
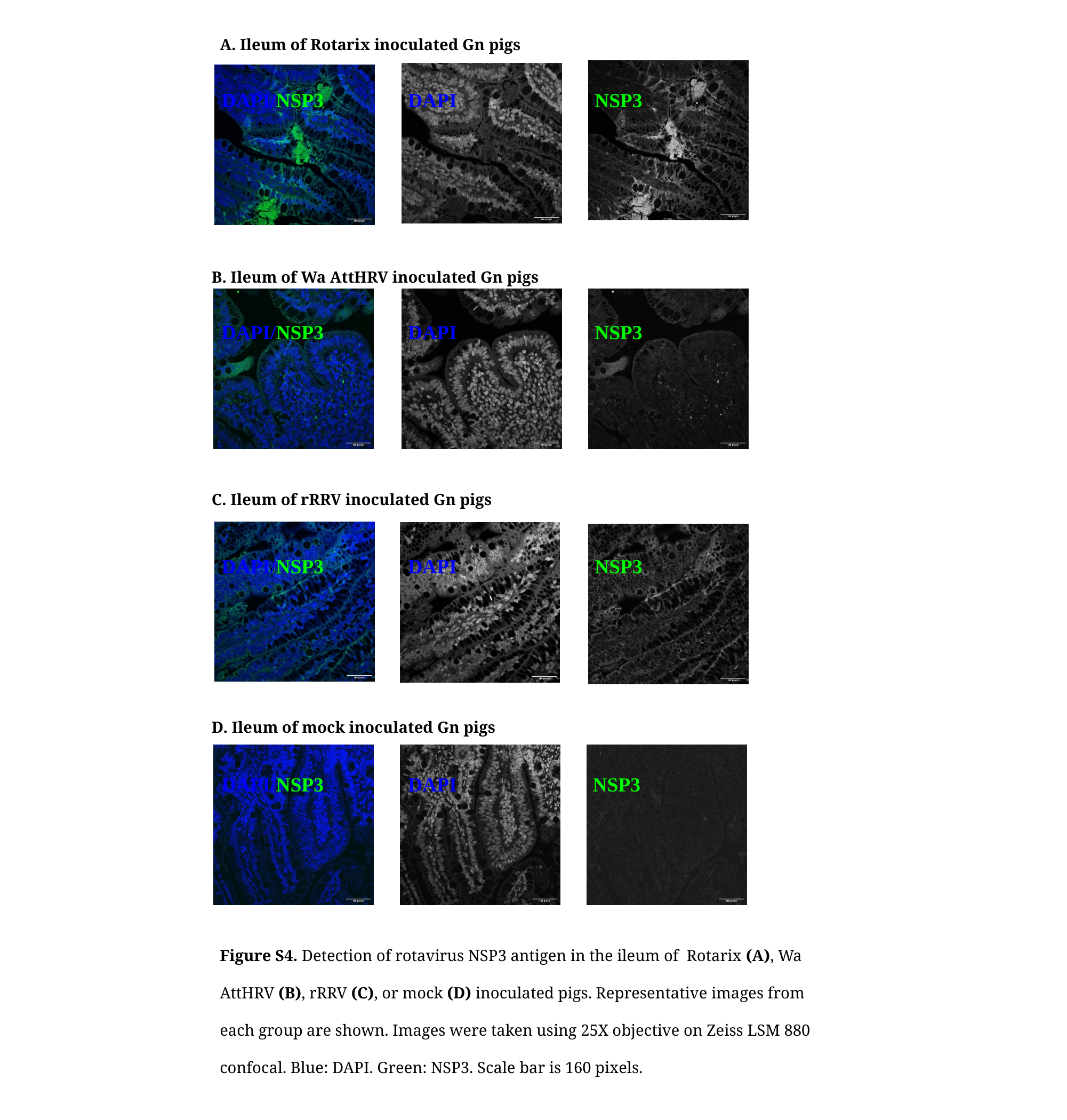

A. Ileum of Rotarix inoculated Gn pigs
DAPI/NSP3
DAPI
NSP3
B. Ileum of Wa AttHRV inoculated Gn pigs
DAPI/NSP3
DAPI
NSP3
C. Ileum of rRRV inoculated Gn pigs
DAPI/NSP3
DAPI
NSP3
D. Ileum of mock inoculated Gn pigs
DAPI/NSP3
DAPI
NSP3
Figure S4. Detection of rotavirus NSP3 antigen in the ileum of Rotarix (A), Wa AttHRV (B), rRRV (C), or mock (D) inoculated pigs. Representative images from each group are shown. Images were taken using 25X objective on Zeiss LSM 880 confocal. Blue: DAPI. Green: NSP3. Scale bar is 160 pixels.
